# Chordae Tendinea Rupture and Pulmonary Valve Vegetation in Pediatric Endocarditis: A Rare Condition

**DOI:** 10.7759/cureus.57694

**Published:** 2024-04-05

**Authors:** Nayakawadi Akhil, Amar Taksande, Revat J Meshram

**Affiliations:** 1 Pediatrics, Jawaharlal Nehru Medical College, Datta Meghe Institute of Higher Education & Research, Wardha, IND

**Keywords:** chordae tendinea rupture, surgical intervention, multidisciplinary management, rheumatic carditis, pulmonary valve vegetation, pediatric endocarditis

## Abstract

Infective endocarditis (IE) is a severe yet rare condition in pediatric patients, often presenting with nonspecific symptoms, which can complicate diagnosis. Chordae tendinea rupture and pulmonary valve involvement are uncommon complications of IE, warranting timely recognition and management to prevent further morbidity and mortality. We present a case of a nine-year-old male child with a rare presentation of endocarditis complicated by chordae tendinea rupture and pulmonary valve vegetation. The child presented with a one-month history of abdominal pain, dyspnea, edema, and cough. Initial investigations revealed severe mitral regurgitation (MR) and tricuspid regurgitation (TR), chordae tendinea rupture, and vegetation on the pulmonary valve. Despite antibiotic therapy, the child's symptoms persisted, necessitating transfer to a tertiary care center for advanced cardiac management. Chordae tendinea rupture is a rare but critical complication of endocarditis, leading to significant valvular dysfunction. Pulmonary valve involvement in endocarditis is relatively uncommon, with most cases involving the mitral and aortic valves. Identifying vegetation on the pulmonary valve underscores the importance of comprehensive echocardiographic evaluation in patients with suspected endocarditis, regardless of valve involvement. Management of pediatric endocarditis involves a multidisciplinary approach, including antibiotic therapy and potential surgical intervention. Despite antibiotic therapy, the child continued to experience fever spikes in this case, indicating a potential need for surgical intervention. In conclusion, this case report highlights the rare presentation of chordae tendinea rupture and pulmonary valve vegetation in pediatric endocarditis. Timely diagnosis and appropriate management, including antibiotic therapy and potential surgical intervention, are essential for optimizing outcomes in affected children.

## Introduction

Infective endocarditis (IE) is a relatively uncommon but potentially life-threatening condition, particularly in pediatric patients. It is characterized by microbial infection of the endocardial surface of the heart, typically affecting the valves, although it can also involve other cardiac structures [1]. The condition is associated with significant morbidity and mortality, and its diagnosis and management pose challenges, especially in pediatric populations where clinical manifestations may be subtle or atypical [2]. Chordae tendinea rupture is a rare complication of IE, occurring when the infective process erodes the chordae tendineae. These fibrous cords anchor the atrioventricular valves to the papillary muscles of the heart. This can lead to valve dysfunction, regurgitation, and hemodynamic compromise [3]. While chordae tendinea rupture is more commonly associated with degenerative valve disease in adults, it can also occur in the setting of IE, particularly in pediatric patients with underlying congenital heart disease [4].

Vegetations, or abnormal growths composed of fibrin, platelets, and microorganisms, are a hallmark of IE. While they most commonly affect the atrioventricular valves, the involvement of other valves, including the pulmonary valve, is less frequent [5]. Pulmonary valve vegetations are rare and may present unique diagnostic and therapeutic challenges, especially in pediatric patients where clinical suspicion may be low. Early recognition and prompt treatment of IE, including appropriate antibiotic therapy and, in some cases, surgical intervention, are essential to prevent complications such as heart failure, embolic events, and valvular dysfunction [6]. However, the diagnosis of IE in pediatric patients can be challenging due to nonspecific clinical manifestations, necessitating a high index of suspicion and a multidisciplinary approach to management [7].

## Case presentation

A nine-year-old male child was brought to the hospital by his father with a chief complaint of abdominal pain persisting for the last month. The pain was acute in onset, gradually progressive, localized to the right hypochondrium, and associated with tenderness. Concurrently, the child had been experiencing increasing breathlessness over the same duration. Initially, he could walk longer distances without difficulty, but over time, he became progressively dyspneic, with the inability to walk even 100 meters without experiencing significant shortness of breath. Furthermore, the child had developed edema, which began in the lower limbs and gradually progressed to involve the upper limbs and face over the past three weeks. Additionally, he had a non-productive cough for the past one to two weeks, not associated with fever or hemoptysis.

**Figure 1:**
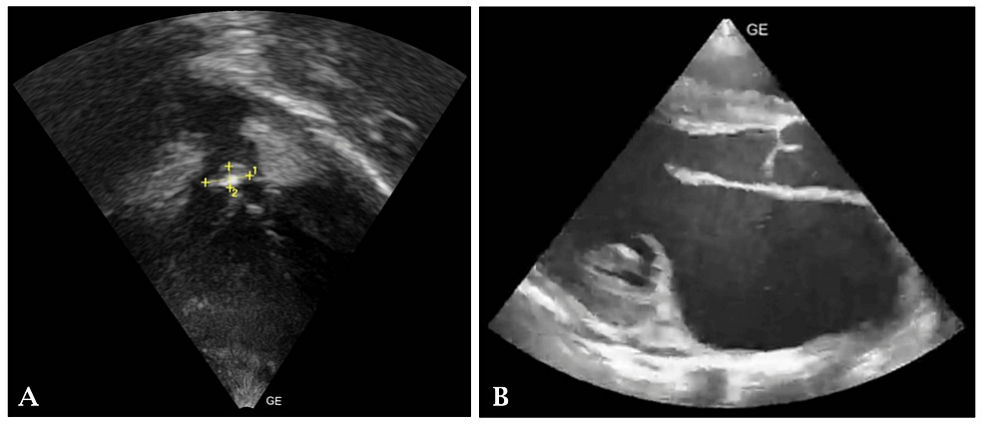
A) Pulmonary vegetation. B) Ruptured chordae tendineae and failed AML AML: anterior mitral leaflet

The father reported that the child's symptoms prompted a visit to a local hospital, where an ultrasound of the abdomen and pelvis was performed, revealing mild to moderate enlargement of the liver (13.3 cm). Subsequently, a 2D echocardiogram was conducted, which demonstrated severe mitral regurgitation (MR), severe tricuspid regurgitation (TR), and an ejection fraction (EF) of 55%, indicative of acute rheumatic carditis (Figure 1). Notably, the echocardiogram revealed dilation of the left and right atrium and a flail anterior mitral leaflet (AML) due to chordae tendinea rupture. Furthermore, vegetation on the pulmonary valve was identified.

Upon admission to the hospital, the child was placed on oxygen support via a non-rebreather mask and initiated on nil by mouth (NBM) status. Intravenous fluids, ceftriaxone, pantoprazole, ondansetron, furosemide, dobutamine, hydrocortisone, nebulization with salbutamol and ipratropium, Omnacortil tablets, ciprofloxacin tablets, and gentamicin injections were administered. Laboratory investigations revealed a hemoglobin level of 10.7 g/dL, total leukocyte count of 25,200 cells/mm³, C-reactive protein level of 24 mg/L, erythrocyte sedimentation rate of 36 mm/hr, positive anti-streptolysin O (ASO) titer, and elevated troponin I level of 152 ng/mL.

Given the elevated total leukocyte count, gentamicin was added to the treatment regimen. However, despite antibiotic therapy, the child continued to experience fever spikes, prompting a change to meropenem and vancomycin. Due to the persistence of symptoms and the need for further cardiac intervention, the child was deemed stable for transfer to a tertiary care center for advanced cardiac management, including the possibility of surgical intervention such as annuloplasty with notochordal repair.

## Discussion

IE is a rare but severe condition in pediatric patients, often presenting with nonspecific symptoms that can complicate diagnosis [8]. Chordae tendinea rupture, although uncommon, can occur as a complication of IE, leading to significant valvular dysfunction [9]. Additionally, vegetation on the pulmonary valve is relatively rare compared to other valve sites [10]. This case report underscores the importance of promptly recognizing and managing these rare complications to prevent further morbidity and mortality in affected children. The diagnosis of IE in pediatric patients can be challenging due to the variability in clinical presentation and nonspecific symptoms [8]. In the presented case, the child initially presented with abdominal pain, dyspnea, edema, and cough, which are not typical symptoms associated with endocarditis. However, further investigations, including echocardiography, revealed severe MR and TR, chordae tendinea rupture, and vegetation on the pulmonary valve, consistent with IE.

Chordae tendinea rupture is a rare complication of endocarditis that can result in acute valvular dysfunction, leading to hemodynamic compromise [9]. The rupture of chordae tendineae can cause flail leaflets, as observed in this case, further exacerbating valvular regurgitation and compromising cardiac function. The presence of such mechanical complications underscores the need for prompt diagnosis and intervention to prevent adverse outcomes. Pulmonary valve involvement in endocarditis is relatively uncommon, with most cases involving the mitral and aortic valves [9]. In this case, identifying vegetation on the pulmonary valve highlights the importance of comprehensive echocardiographic evaluation in patients with suspected endocarditis, regardless of valve involvement. Early vegetation detection is crucial for initiating appropriate antibiotic therapy and preventing embolic events and further valve damage. The management of pediatric endocarditis involves a multidisciplinary approach, including antibiotic therapy and, in some cases, surgical intervention [11]. Antibiotic therapy aims to eradicate the infective organism and prevent systemic complications. At the same time, surgical intervention may be necessary to address mechanical complications such as chordae tendinea rupture or persistent valvular dysfunction despite medical therapy. Despite antibiotic therapy, the child continued to experience fever spikes in this case, indicating a potential need for surgical intervention.

## Conclusions

In conclusion, this case report sheds light on the rare but significant presentation of chordae tendinea rupture and pulmonary valve vegetation in pediatric endocarditis. The complexity of this case underscores the challenges in diagnosing and managing IE in children, given its varied clinical manifestations. Prompt recognition of mechanical complications such as chordae tendinea rupture is vital for timely intervention and optimal outcomes. Additionally, identifying pulmonary valve involvement emphasizes the importance of comprehensive echocardiographic evaluation in suspected cases of endocarditis. Despite aggressive antibiotic therapy, the persistence of symptoms in this case highlights the potential need for surgical intervention in selected patients. This case serves as a reminder of the importance of a multidisciplinary approach involving pediatricians, cardiologists, and cardiac surgeons to ensure comprehensive care for children with endocarditis. Further research and clinical studies are warranted to elucidate optimal management strategies for such complex cases and improve outcomes in pediatric patients with IE.
